# Phylogenetic analyses of *Salmonella* detected along the broiler production chain in Trinidad and Tobago

**DOI:** 10.1016/j.psj.2022.102322

**Published:** 2022-11-10

**Authors:** Anisa S. Khan, Rian E. Pierneef, Narjol Gonzalez-Escalona, Meghan Maguire, Karla Georges, Woubit Abebe, Abiodun A. Adesiyun

**Affiliations:** ⁎ School of Veterinary Medicine, Faculty of Medical Sciences, University of the West Indies, St. Augustine, Trinidad and Tobago; † Agricultural Research Council-Biotechnology Platform, Pretoria 0110, South Africa; ‡ Division of Microbiology, Office of Regulatory Science, Center for Food Safety and Applied Nutrition, Food and Drug Administration, College Park, MD, USA; § Department of Pathobiology, Center for Food Animal Health, Food Safety and Food Defense, Tuskegee University, College of Veterinary Medicine, Tuskegee, AL 36088, USA; # Department of Production Animal Studies, Faculty of Veterinary Science, University of Pretoria, Pretoria 0110, South Africa

**Keywords:** Salmonella, broiler production chain, WGS, phylogeny, Trinidad and Tobago

## Abstract

This study was conducted to determine the phylogenies of *Salmonella* strains isolated from cross-sectional studies conducted at hatcheries, broiler farms, processing plants, and retail outlets (broiler production chain) in Trinidad and Tobago over 4 yr (2016–2019). Whole-genome sequencing (**WGS**) was used to characterize *Salmonella* isolates. Core genome phylogenies of 8 serovars of public health significance were analyzed for similarities in origin and relatedness. In addition, *Salmonella* strains isolated from human salmonellosis cases in Trinidad were analyzed for their relatedness to the isolates detected along the broiler production chain. The common source of these isolates of diverse serovars within farms, within processing plants, between processing plants and retail outlets, and among farm-processing plant-retail outlet continuum was well-supported (bootstrap value >70%) by the core genome phylogenies for the respective serovars. Also, genome analyses revealed clustering of *Salmonella* serovars of regional (intra-Caribbean) and international (extra-Caribbean) origin. Similarly, strains of *S.* Enteritidis and *S.* Infantis isolated from human clinical salmonellosis in 2019 from Trinidad and Tobago clustered with our processing plant isolates recovered in 2018. This study is the first phylogenetic analysis of *Salmonella* isolates using WGS from the broiler industry in the Caribbean region. The use of WGS confirmed the genetic relatedness and transmission of *Salmonella* serovars contaminating chickens in broiler processing, and retailing in the country, with zoonotic and food safety implications for humans.

## INTRODUCTION

The recent technological advancements and declining costs of next-generation sequencing tools such as Whole-genome sequencing (**WGS**) have increased the efficiency and resolution of detection, characterization, and surveillance of infectious pathogens of public health importance. The vast potential of WGS in the surveillance of infectious diseases (Reuter et al., 2013; Hasman et al., 2014) has been demonstrated in many studies (Hendriksen et al., 2011; Mellmann et al., 2011; Allard et al., 2012). A more effective and rational approach for preventing microbial threats is essential at the global level due to increases in the food trade, population density and movement, and consumption habits which have increased the risk of contracting foodborne diseases and their potential global spread. To harness the full potential of WGS, the global microbial identifier (**GMI**) initiative was developed as a shared global database of genomes to diagnose infectious diseases in humans and animals, identify microorganisms in food and the environment, as well as track and trace microbial agents (Wielinga et al., 2017). Sharing sequencing data within the global scientific community supports the mission of public health institutions and the One Health concept. Furthermore, this approach facilitates early recognition and investigation of international outbreaks in addition to providing invaluable information to clinicians, veterinarians, environmental scientists, as well as policymakers, regulators, and industry.

While pulse-field gel electrophoresis (**PFGE**) remains the primary subtyping method in PulseNet, other than Canada and the United States, no other countries since 2005 shared molecular surveillance data this way (PulseNet) in real-time until the advent of WGS (Gerner-Smidt et al., 2019). WGS has now been deemed the gold standard for molecular surveillance of foodborne diseases due to the wealth of information available that exceeds previous traditional phenotypic and genotypic tests, such as serovar, pathotype, virulence profiles, antimicrobial resistance, and plasmid content (Deng et al., 2016). However, it is pertinent to note that organizational challenges such as building appropriate informatics systems, software, and network infrastructure to manage, analyze, transmit, and harmonize them with laboratory and program operations exist (Gwinn et al., 2017). Also, the extent of public availability of information to other health agencies, researchers, and the public are further limitations to the use of WGS (Gwinn et al., 2017).

The risk of salmonellosis exists along the farm-to-fork continuum posing a threat to public health (Collineau et al., 2020). Therefore, it is vital to utilize the molecular analysis of *Salmonella* strains to investigate their distribution, transmission, and characteristics. Serotyping using traditional techniques is the predominant practice to determine serovars, and the relatedness of *Salmonella* strains from foods, animals, environment, and disease outbreaks in the Caribbean (Rampersad et al., 1999; Indar-Harrinauth et al., 2001; Adesiyun et al., 2009; Guyomard-Rabenirina et al., 2019; Kumar et al., 2019; Buteux et al., 2020). PFGE has previously been used to investigate the diversity of *Salmonella* strains isolated in the poultry industry (Kumar et al., 2020) and human gastroenteritis in the region (Adesiyun et al., 2000). Our recent publication characterized *Salmonella* detected along the broiler production chain using WGS, the first study conducted in the Caribbean region (Khan et al., 2022b). However, published phylogenetic studies using WGS of *Salmonella* isolated from the broiler industry in Trinidad and Tobago and the Caribbean region at large are non-existent. The high molecular resolution offered by WGS compared with PFGE allows for more accurate identification and characterization of bacterial strains (Salipante et al., 2015). Surveillance systems that integrate public health, animal health, and food production are optimal for detecting, investigating and solving infections commonly transmitted through food.

Therefore, the primary objective of this study was to investigate the genetic relatedness of *Salmonella* serovars recovered from the broiler industry in Trinidad and Tobago using WGS.

## MATERIALS AND METHODS

### Sample Selection

A total of 137 isolates of *Salmonella* selected for use in this study originated from studies conducted on hatcheries and broiler farms (Khan et al., 2022a), processing plants (Khan et al., 2021), and retail outlets (pluck shops and supermarkets) (Khan et al., 2018) over 4 yr. The following is a summary of the number of isolates included from earlier studies: hatcheries (n = 10), farms (n = 20), processing plant (n = 52), and retail outlets (n = 55). Five additional human clinical isolates of *Salmonella* belonging to serovars *S.* Enteritidis and *S*. Infantis, obtained from the Caribbean Public Health Agency (**CARPHA**), Trinidad and Tobago, were included in our panel of isolates subjected to WGS*.* These human strains were included to compare *Salmonella* strains detected along the broiler production chain to human salmonellosis, since to date, there has been no association between the two. The selected isolates represent *Salmonella* strains recovered from various samples, serovars, and enrichment (selective and non-selective) broths and agar used to isolate the pathogen.

### DNA Extraction and Sequencing

DNA was extracted using both Maxwell RSC cultured cells DNA kit with a Maxwell RSC instrument (Promega, Madison, WI) and DNeasy Blood & Tissue Kit (Qiagen, Germany) following the manufacturer's protocols for Gram-negative bacteria with additional RNase treatment. DNA concentrations were measured with a Qubit fluorometer (Life Technologies, Carlsbad, CA), standardized to 0.2 ng/µL, and the samples were stored at 4°C before library preparation.

Whole-genome sequencing (WGS) of the *Salmonella* isolates was performed by the OIE Reference Laboratory for Salmonellosis, National Microbiology Laboratory of Guelph (NLM@Guelph) of the Public Health Agency of Canada (**PHAC**), and the Food and Drug Administration (**FDA**): Center for Food Safety and Applied Nutrition genomics laboratory (**FDA-CFSAN**) and Center for Veterinary Medicine (**FDA-CVM**), Maryland, USA. The WGS data was generated on an Illumina MiSeq using 2 × 250 bp paired-end chemistry (Illumina, San Diego, CA) and 2 × 300 bp paired-end reads according to the manufacturer's instructions, at 50–150X coverage. According to the manufacturer's instructions, the libraries were constructed using 100 ng of genomic DNA using the Illumina DNA Prep (M) Tagmentation kit (Illumina) and 1 ng of genomic DNA using the Nextera XT kit (Illumina).

### Genomic Data Analysis and in Silico Determination of Genetic Elements

Quality control, including adapter removal of the raw data, was done using BBDuk (v.37.90; https://jgi.doe.gov/data-and-tools/bbtools/bb-tools-user-guide/bbduk-guide/; sourceforge.net/projects/bbmap/). SPAdes v.3.12.0 (Bankevich et al., 2012) was used to create a de novo assembly of each isolate. Only contigs longer than 500 bp were retained for further analysis. Serovar prediction was made using the command-line version of SISTR (Yoshida et al., 2016) (Version: sistr_cmd v.1.1.1).

### Determination of Phylogeny

Maximum likelihood phylogenies of all serovars (i.e., serovars comprising 4 or more isolates) from this study, in addition to reference genomes (completely closed genomes) for each serovar, downloaded from NCBI, were calculated using core genome alignment. Reference genomes used for each serovar was as follows: Albany_strain_R17.2117 (Accession: NZ_CP063330), Anatum_strain_ATCC_BAA_1592 (Accession: CP007531), Enteritidis_strain_P125109 (Accession: NC_011294), Infantis_strain_119944 (Accession: NZ_CP047881), Javiana_strain_CFSAN001992 (Accession: NC_020307), Kentucky_strain_CVM_30177(Accession: NZ_CP051346), Manhattan_strain_SA20084699 (Accession: NZ_CP022497), Schwarzengrund_strain_CVM_30168 (Accession: NZ_CP051350), and Senftenberg_strain_CVM_34514 (Accession: NZ_CP051329). Other publicly available *Salmonella* strains were randomly selected, downloaded, and calculated using core genome alignment using the following criteria for each serovar: detected along the broiler production chain, originating from the Caribbean, South America, North America, Europe, or Asia, as well as the availability of the raw sequences on the NCBI database (Supplementary Tables 1–9). It was important to include other publicly available strains in our study due to the worldwide trade of poultry and more so because this is the first phylogenetic study focusing on *Salmonella* detected from the broiler industry in the region. As such, any comparison with international/regional strains was deemed valuable.

Core Genome Phylogeny was inferred by creating a core genome alignment with Roary (Page et al., 2015) and using the resulting alignment in IQ-TREE v.2.1.2 (Minh et al., 2020) to produce a tree file and 1,000 bootstrap pseudo-replications. The tree file was visualized with the R package ggtree (Yu et al., 2016). Bootstrap values ≥70% were considered well-supported/reliable and were indicated by a black dot on the node in the respective figures.

## RESULTS

### Core Genome Phylogenies

#### *S*. Albany

**Figure 1:**
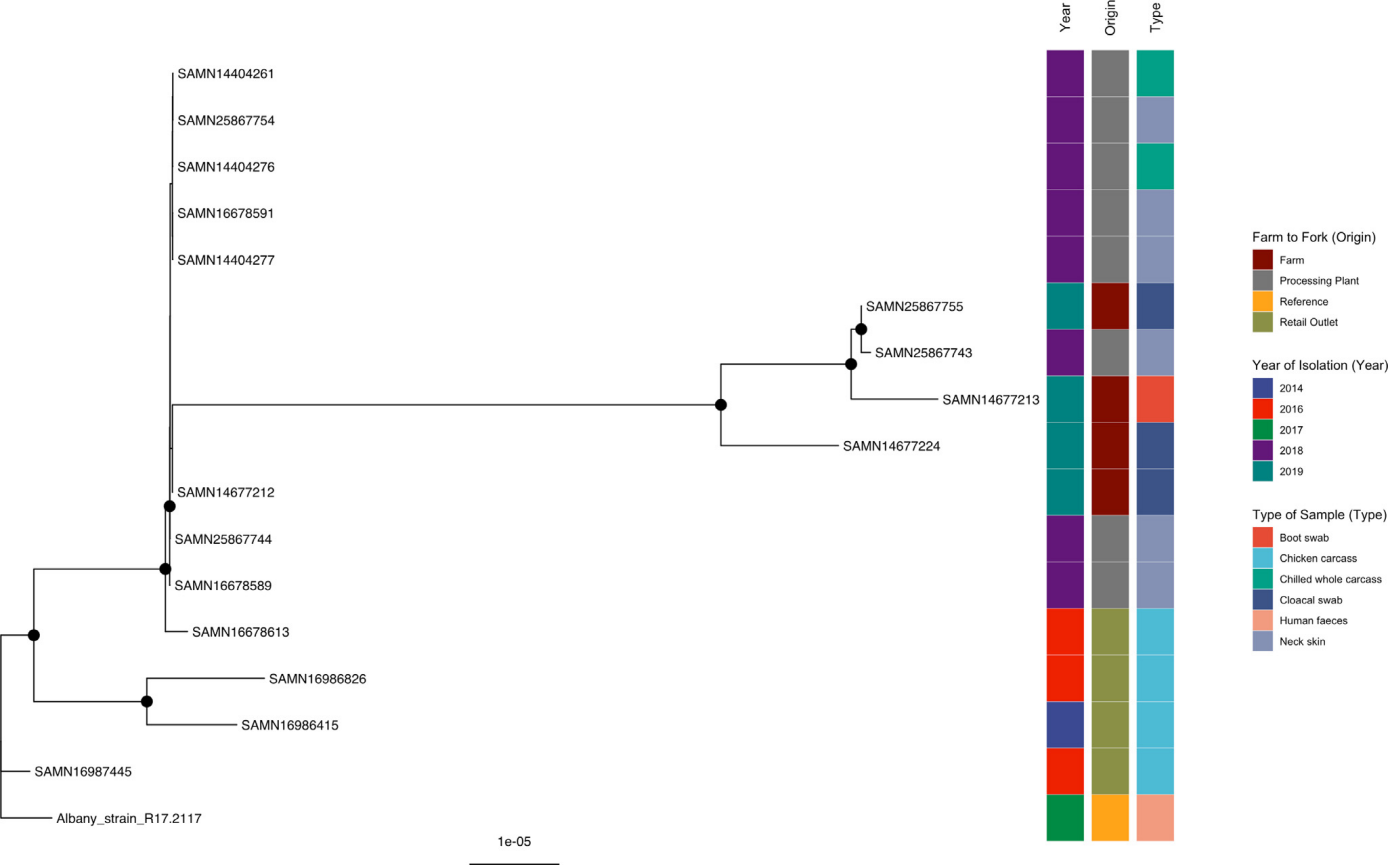
Phylogenetic analysis of *S.* Albany. Maximum-likelihood phylogeny of *S.* Albany is shown, calculated using a core genome alignment with IQ-TREE v.2.1.2 and 1,000 bootstraps specified. A black dot on the node indicates bootstrap values >70%.

Strain SAMN16678613, isolated in 2016 from a chicken carcass sold at a retail outlet in the current study, was grouped with all the other isolates collected in 2018 and 2019 from processing plants and farms, respectively (Figure 1, Supplementary Table 1), supported by a bootstrap value of 100%. SAMN14677224 isolated from broiler farm C (Processor A; cloacal swab) in 2019 was grouped with SAMN14677213 (boot swab taken at farm F operated by Processor B in 2019), SAMN25867743 (neck skin collected at processing plant B in 2018), and SAMN25867755 (cloacal swab collected at a farm A operated by Processor B in 2019) and was also supported by a bootstrap value of 100%. SAMN16678613, detected in 2016 from a retail outlet clustered to the previously mentioned isolates, highlighting the linkages of *S.* Albany isolated at different sampling levels in this study (retail outlet, various farms, and processing plants). Publicly available isolates, SAMN16986415 and SAMN16986826, detected at retail outlets in China in 2014 and 2016, respectively, were clustered to all the Albany isolates from various types of samples and origin isolated between 2016 and 2019 in the current study. Two strains included in this tree were isolated in Taiwan from a retail outlet (SAMN16987445) and human feces (reference strain; Albany_strain_R17.2117) from 2016 and 2017, respectively not clustered together nor to any of the isolates.

#### *S*. Anatum

**Figure 2:**
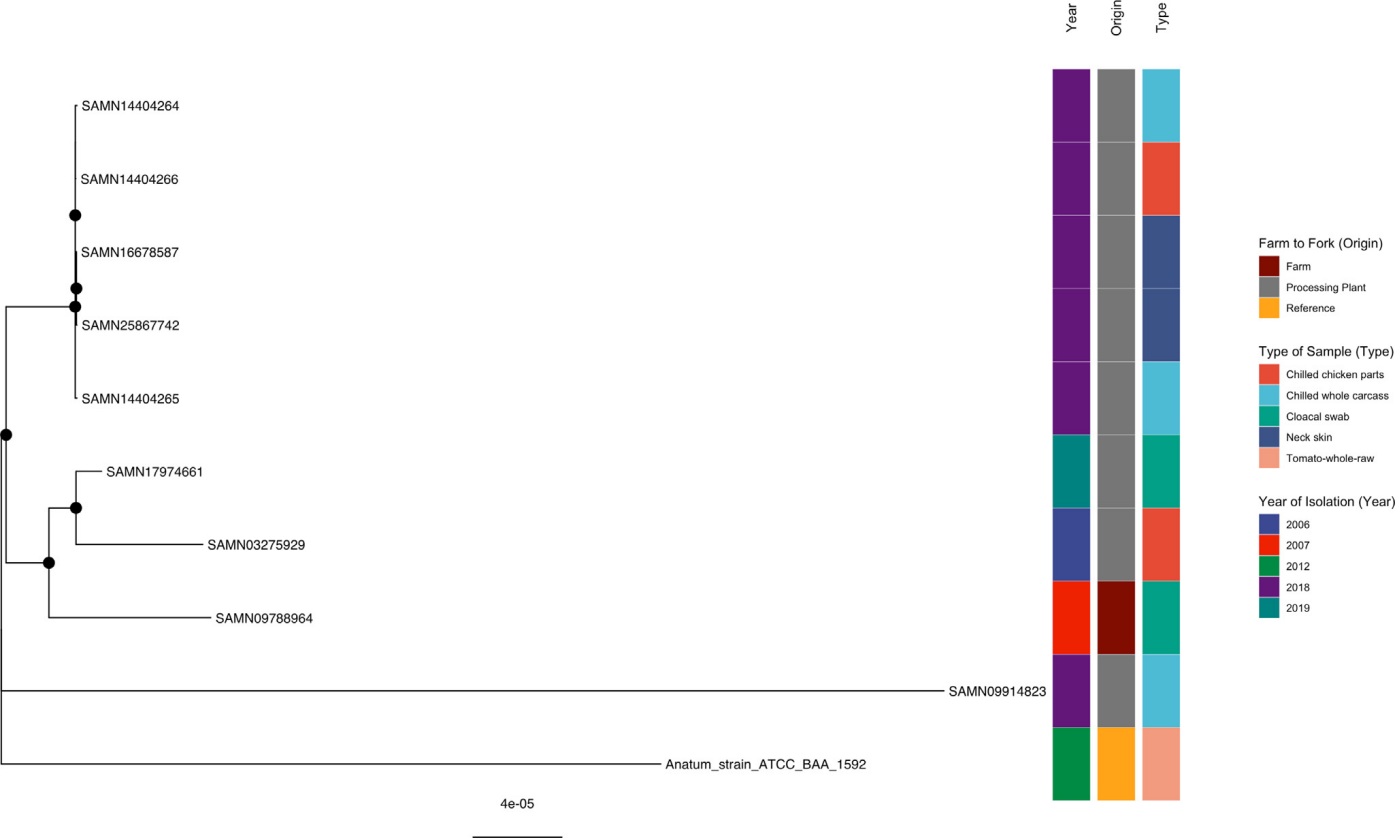
Phylogenetic analysis of *S.* Anatum. Maximum-likelihood phylogeny of *S.* Anatum is shown, calculated using a core genome alignment with IQ-TREE v.2.1.2 and 1,000 bootstraps specified. A black dot on the node indicates bootstrap values >70%.

All the *S*. Anatum isolates recovered at processing plants in 2018 from chilled whole/chicken parts, and neck skin samples (Supplementary Table 2) were clustered together and well-supported by a bootstrap value of 100% (Figure 2). All five isolates (SAMN25867742, SAMN16678587, SAMN14404266, SAMN14404264, and SAMN14404265) were detected on the same sampling day and at the same processing plant B. Two publicly available isolates, SAMN03275929 (USA processing plant in 2006) and SAMN09788964 (Taiwan, farm in 2007), were clustered with isolate SAMN17974661 (USA processing plant in 2019), and all the processing plant isolates detected in 2018 with supporting bootstrap values. Reference strain Anatum_strain_ATCC_BAA_1592 (raw tomato, 2012, USA) and publicly sourced isolate SAMN09914823 (Taiwan processing plant isolate, 2018) exhibited no clustering to one another as well as to the isolates in the current study.

#### *S*. Enteritidis

**Figure 3:**
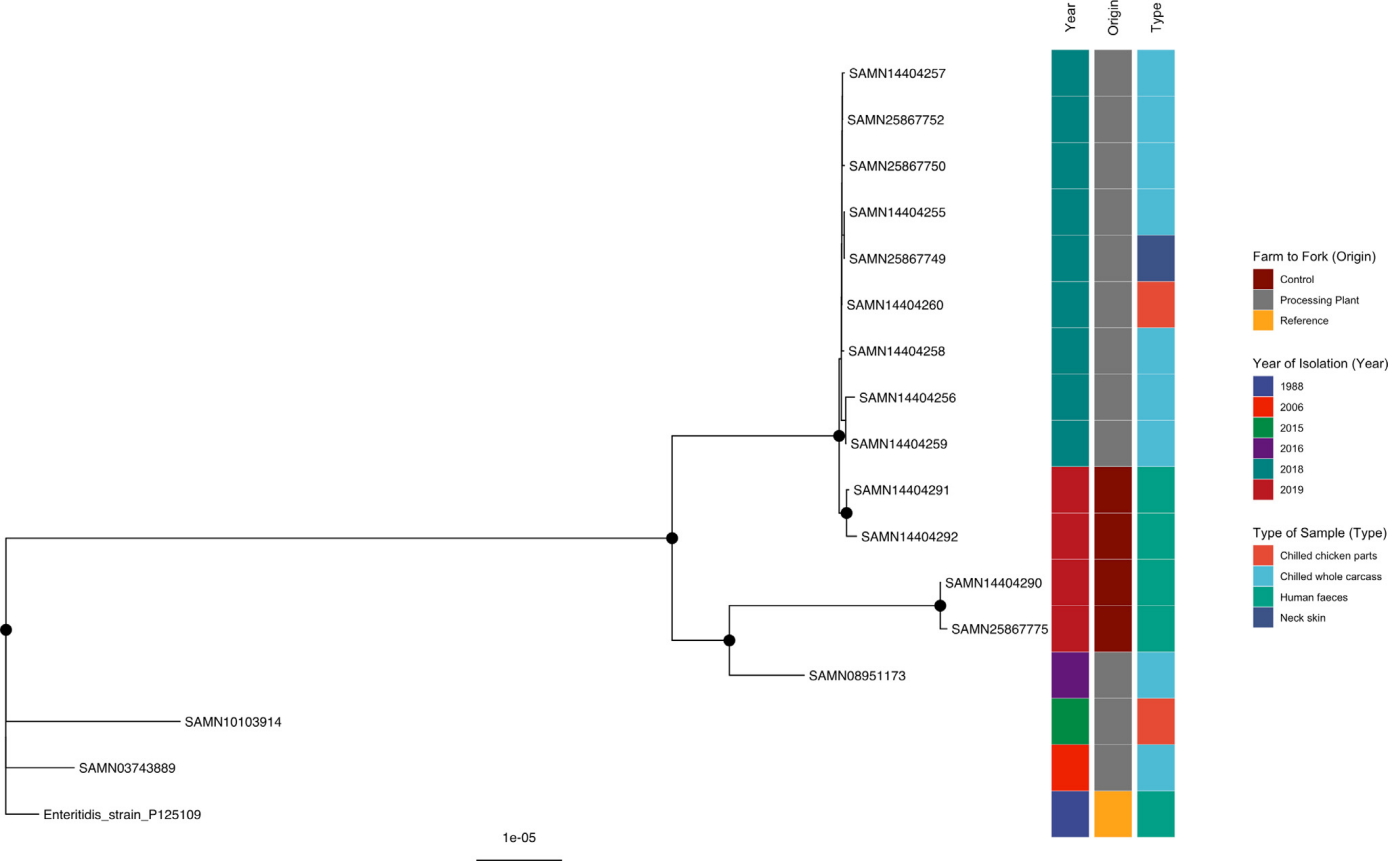
Phylogenetic analysis of *S.* Enteritidis*.* Maximum-likelihood phylogeny of *S.* Enteritidis is shown, calculated using a core genome alignment with IQ-TREE v.2.1.2 and 1,000 bootstraps specified. A black dot on the node indicates bootstrap values >70%.

The four human clinical isolates of *Salmonella* from Trinidad and Tobago isolated in 2019 exhibited clustering to one another, as well as to all processing plant isolates recovered in 2018 (Supplementary Table 3) from the chilled whole carcass, chilled chicken parts, and neck skin in the current study, well-supported by bootstrap values. Within processing plant isolates, 2 clustered strains were detected during the same sampling visit to Plant A (SAMN25867749 and SAMN14404255) and Plant C (SAMN25867750 and SAMN14404256) (Figure 3). Also, four strains were detected at Plant D on 2 separate sampling days: strains SAMN25867752 and SAMN14404259 on sampling d 1 and strains SAMN14404260 and SAMN14404258 on d 2.

#### *S*. Infantis

**Figure 4:**
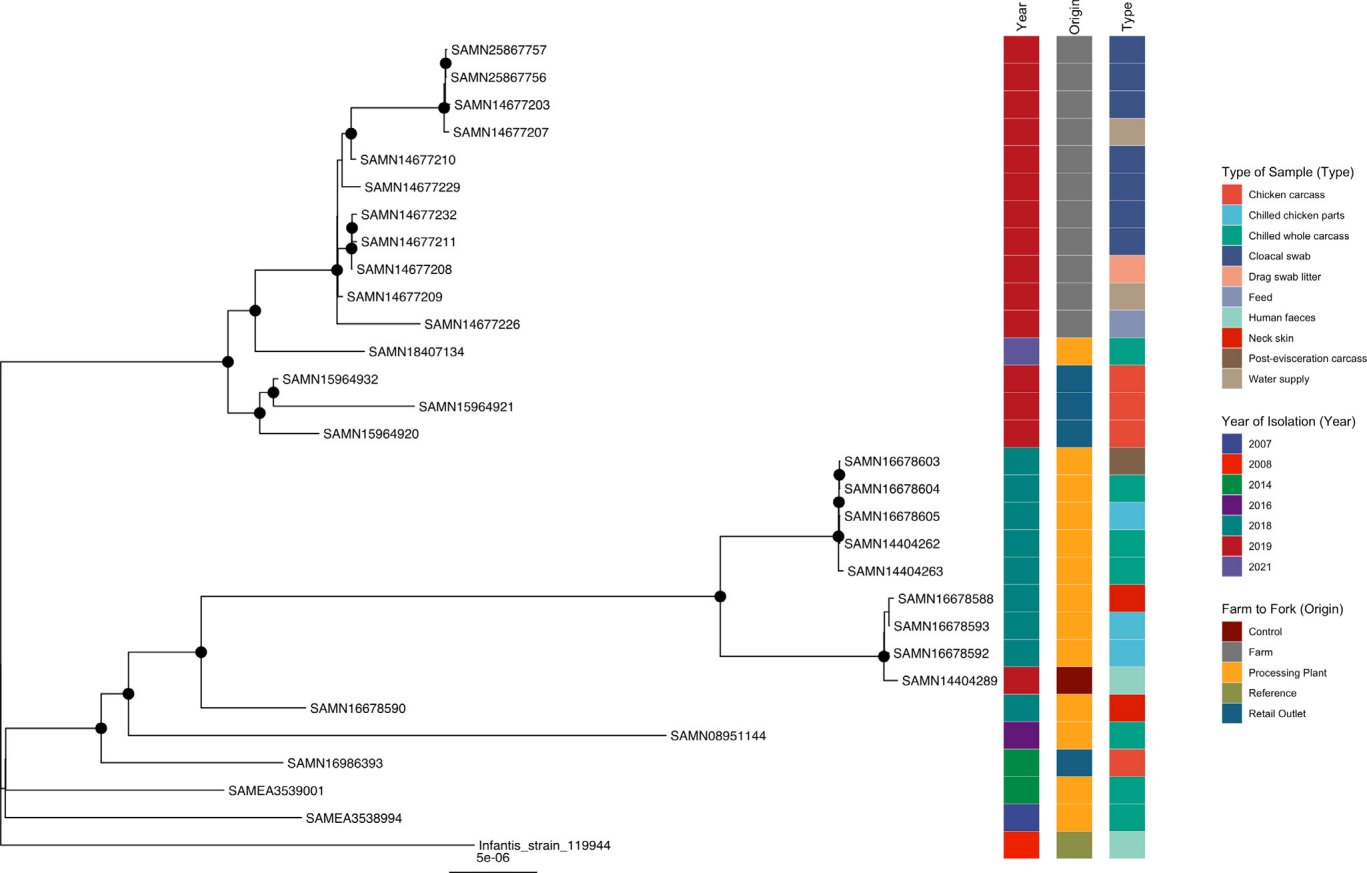
Phylogenetic analysis of *S.* Infantis*.* Maximum-likelihood phylogeny of *S.* Infantis is shown, calculated using a core genome alignment with IQ-TREE v.2.1.2 and 1,000 bootstraps specified. A black dot on the node indicates bootstrap values >70%.

Two distinct clusters were observed. In the first, SAMN08951144, isolated from a Brazilian processing plant in 2016 (Supplementary Table 4), showed well-supported clustering with isolates from neck skin (SAMN16678590 and SAMN16678588) and chilled chicken parts (SAMN16678592 and SAMN16678593) isolates, all from processing plant B (same sample day) in the current study, as well as the human clinical isolate of *Salmonella* recovered (SAMN14404289) in 2019 in the present study (Figure 4). SAMN14404263 isolated from chilled whole carcass exhibited well-supported clustering with the other chilled whole carcass (SAMN16678604 and SAMN14404262), chilled chicken parts (SAMN16678605), and post-evisceration carcass (SAMN16678603), all isolated at Plant A on the same sampling day. The latter 4 isolates were clustered together with a bootstrap value of 97 %. Our farm isolates were clustered with three isolates detected at retail outlets in Barbados and an isolate detected at a processing plant in the United States (SAMN18407134).

In the second cluster, well-supported clustering existed among the farm isolates of *Salmonella,* which originated from cloacal swabs, water supply, and drag swabs of litter in our study. Clustering between the drag swab isolate (SAMN14677208, detected at Farm U, operated by Processor B) and cloacal swab samples (SAMN14677211 and SAMN14677232, detected at Farm C, operated by Processor A&C) was observed. There was also clustering with the water supply sample (SAMN14677207, Farm J, Processor B) and 2 cloacal swab samples (SAMN25867756 and SAMN25867757, Farm K, Processor B), lineages supported by a bootstrap value of 88%. Additionally, clustering was observed among the cloacal swab isolates detected at 2 farms operated by Processor B, Farm K (SAMN25867756, SAMN14677229, and SAMN25867757), and Farm A (SAMN14677203 and SAMN14677210).

#### *S*. Javiana

**Figure 5:**
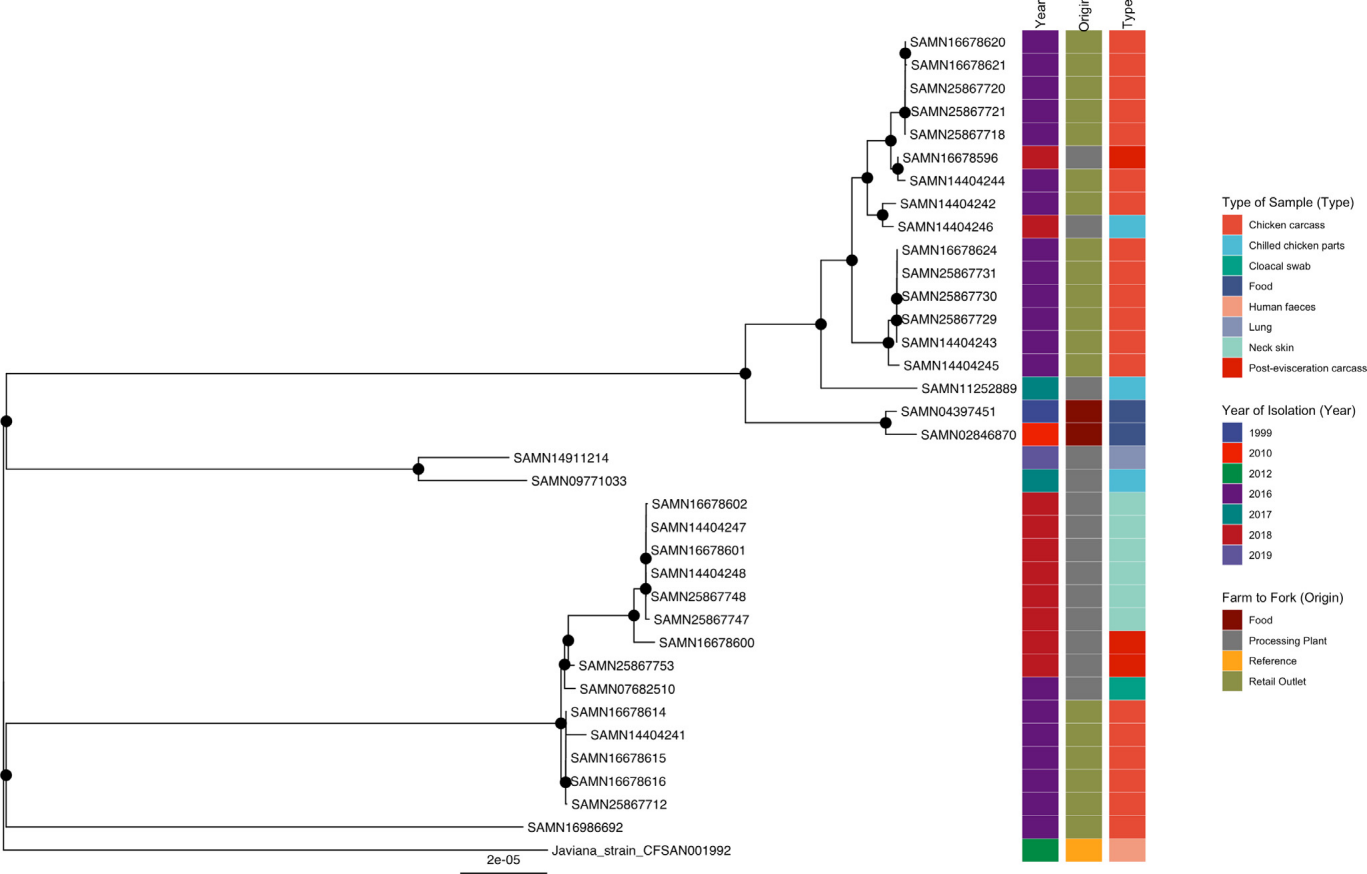
Phylogenetic analysis of *S.* Javiana. Maximum-likelihood phylogeny of *S.* Javiana is shown, calculated using a core genome alignment with IQ-TREE v.2.1.2 and 1,000 bootstraps specified. A black dot on the node indicates bootstrap values >70%.

Two main clusters supported by bootstrap values of 77 and 97%, respectively, were observed in the Javiana phylogenetic tree (Figure 5). Of significance was the well-supported clustering of SAMN16986692, a publicly available isolate (Supplementary Table 5) detected in retail outlets in China in 2016 with retail outlet isolates, SAMN16678614, SAMN14404241, SAMN16678615, SAMN16678616, and SAMN25867712 all detected at one cottage poultry processor in St. George Central. In addition, this subcluster of retail outlet isolates showed reliable clustering with SAMN07682510, isolated from a processing plant in 2016 in a previous study conducted in Trinidad and Tobago, and most (8/10; 80%) of our 2018 processing plant isolates (Plants A and D). Further analyses of our processing plant isolates showed reliable clustering amongst the Javiana isolates detected on all (6/6) neck samples detected at Plant A on the same sample day.

In the second main cluster, isolates SAMN09771033 and SAMN14911214 recovered from processing plants in 2017 and 2019 (both from the United States) exhibited reliable clustering with SAMN11252889 isolated at a Peruvian processing plant in 2017. There was evidence of well-supported clustering of this Peruvian isolate with all the 2016 isolates from retail outlets in the current study. A 2016 retail outlet isolate, SAMN14404242 (detected in the county of St. George East), and a 2018 processing plant isolate, SAMN14404246 (Plant B), were grouped, supported by a bootstrap value of 100%. Isolates SAMN16678596 (Plant B) and SAMN14404244 (cottage processor in the county of Nariva/Mayaro) were also grouped and supported by a bootstrap value of 100%.

Reference strain Javiana_strain_CFSAN001992 isolated from human feces in the United States (2012) exhibited no clustering to any of the isolates included in the tree.

#### *S*. Kentucky

**Figure 6:**
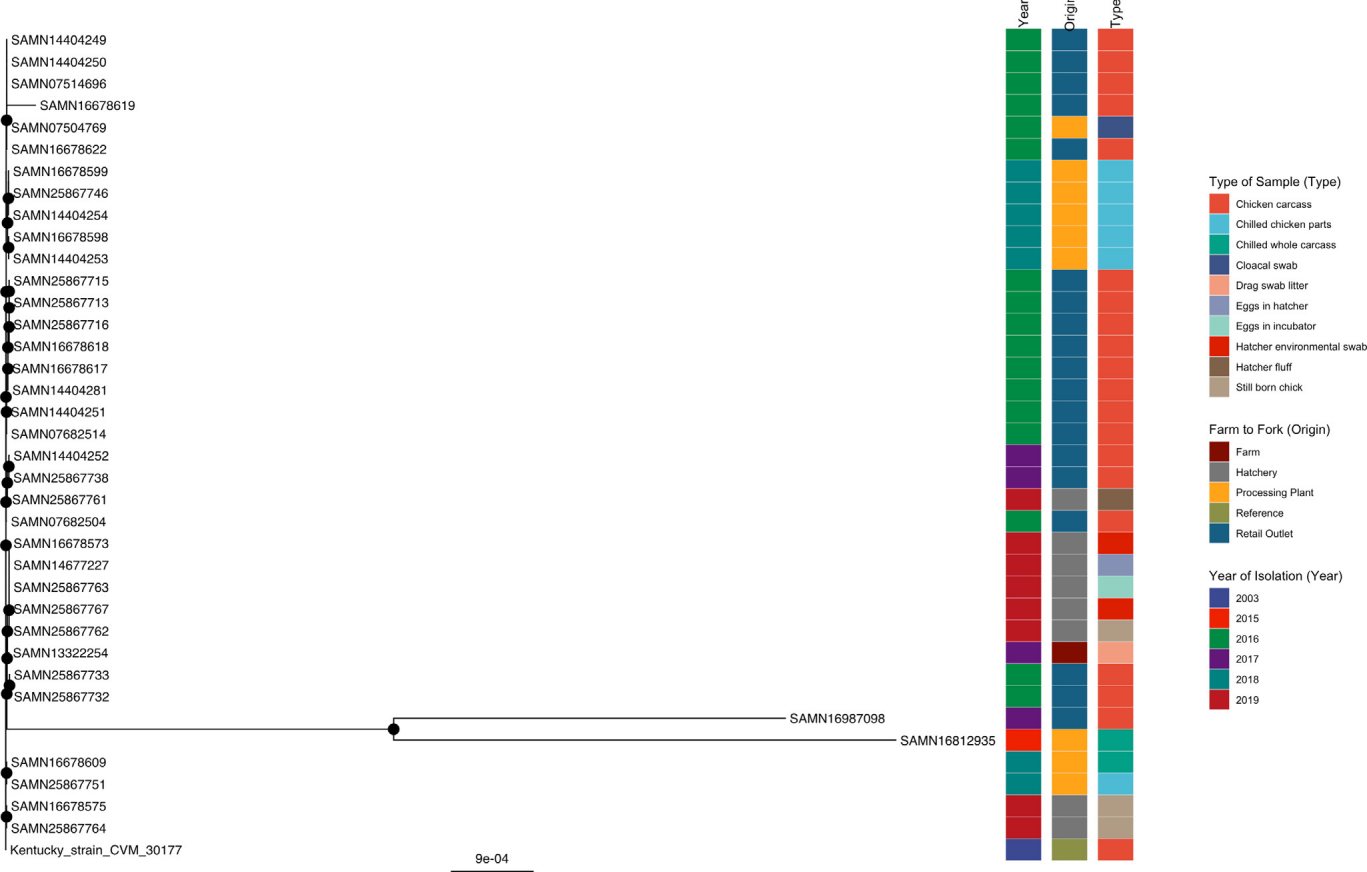
Phylogenetic analysis of *S.* Kentucky. Maximum-likelihood phylogeny of *S.* Kentucky is shown, using a core genome alignment with IQ-TREE v.2.1.2 and 1,000 bootstraps specified. A black dot on the node indicates bootstrap values >70%.

Among the 38 *S.* Kentucky isolates compared, clustering was observed among all the processing plant isolates, supported by bootstrap values of 100%. SAMN14404281, isolated at a retail outlet in 2016 in this study, exhibited clustering supported by a bootstrap value of 100% with other retail outlet isolates (SAMN16678617, SAMN16678618, SAMN25867716, SAMN25867715, and SAMN25867713), all detected at the same cottage poultry processor in the county of Victoria (Figure 6, Supplementary Table 6). Well-supported clustering was also observed with a 2016 retail outlet isolate from Barbados (SAMN07682504), the retail outlet isolates from 2017 (SAMN25867738 and SAMN14404252, chickens sold at different supermarkets), and a hatchery isolate from 2019 (SAMN25867761, operated by Processor A). An isolate recovered from a litter drag swab sample from a farm in the United States in 2017, SAMN13322254, exhibited well-supported clustering with 100% (5/5) of the hatchery serovar Kentucky isolates (SAMN25867762- stillborn chick, SAMN25867767- hatcher environmental swab, SAMN25867763- eggs in the incubator, SAMN14677227- eggs in hatcher and SAMN16678573- hatcher environmental swab) detected at the hatchery operated by Processor B in 2019. International strains, SAMN16812935 and SAMN16987098, detected in a Brazilian processing plant and Chinese retail outlet, respectively, showed well-supported clustering with the previously mentioned hatchery isolates in the current study, in addition to SAMN13322254 (USA, drag swab litter, 2017). Two retail outlet isolates, SAMN25867732, and SAMN25867733 were detected in county Victoria (same cottage processor). The other 2 Kentucky strains detected at hatcheries were clustered together and well-supported (operated by Processor A) but exhibited no clustering to other members of the tree.

#### *S.* Manhattan

**Figure 7:**
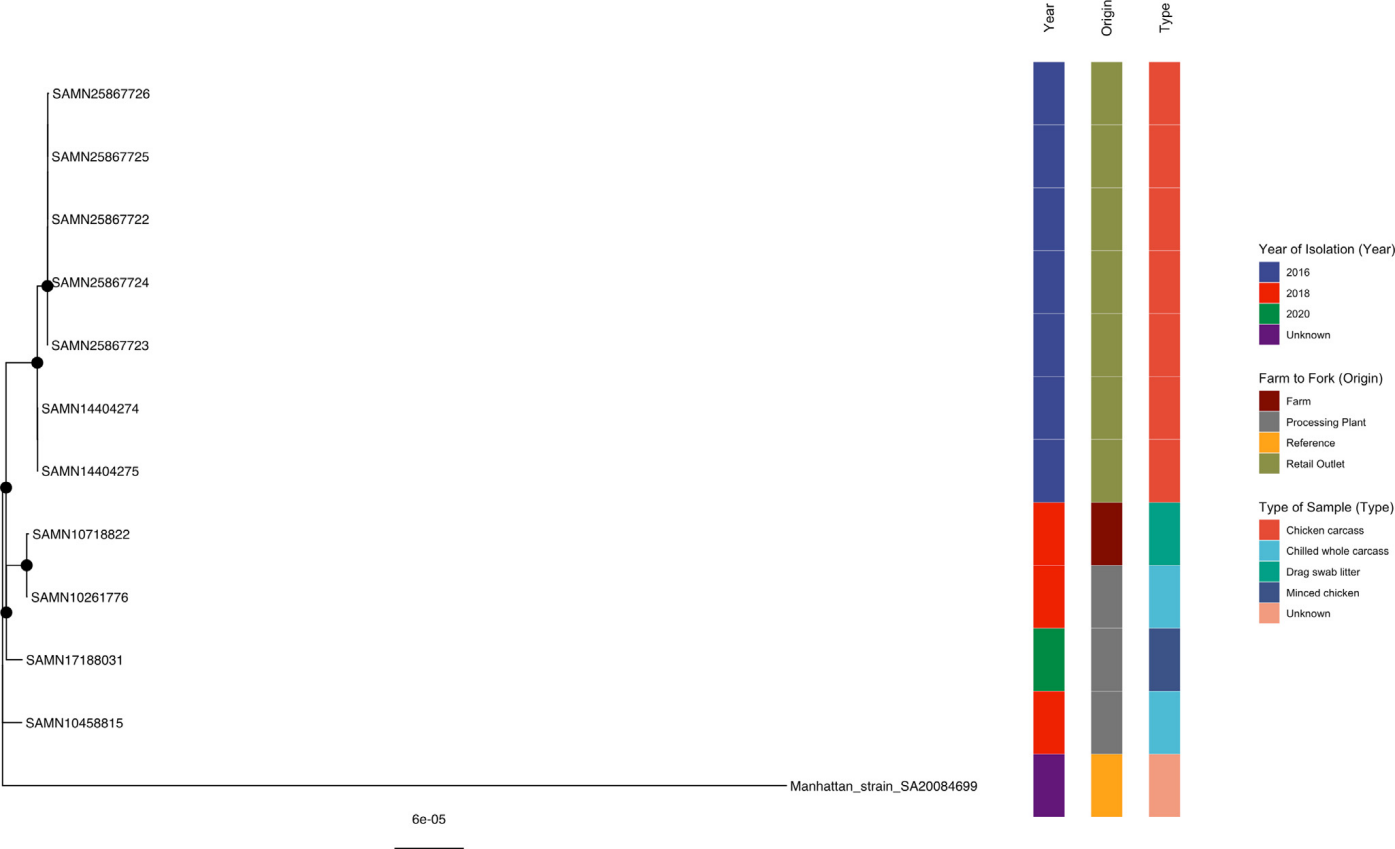
Phylogenetic analysis of *S.* Manhattan. Maximum-likelihood phylogeny of *S.* Manhattan is shown, using a core genome alignment with IQ-TREE v.2.1.2 and 1,000 bootstraps specified. A black dot on the node indicates bootstrap values >70%.

Three international strains (all isolated in the USA) of *Salmonella* isolated from processing plants (SAMN10261776, 2018 and SAMN17188031, 2020) and a farm (SAMN10718822, 2018) were clustered with all 7 retail outlet isolates recovered in 2016, well-supported by a bootstrap value of 100% (Figure 7). In addition, intra-cluster analyses of the seven retail outlet strains in this study exhibited well-supported clustering where all strains were detected from the same pluck shop (Supplementary Table 7).

#### *S*. Schwarzengrund

**Figure 8:**
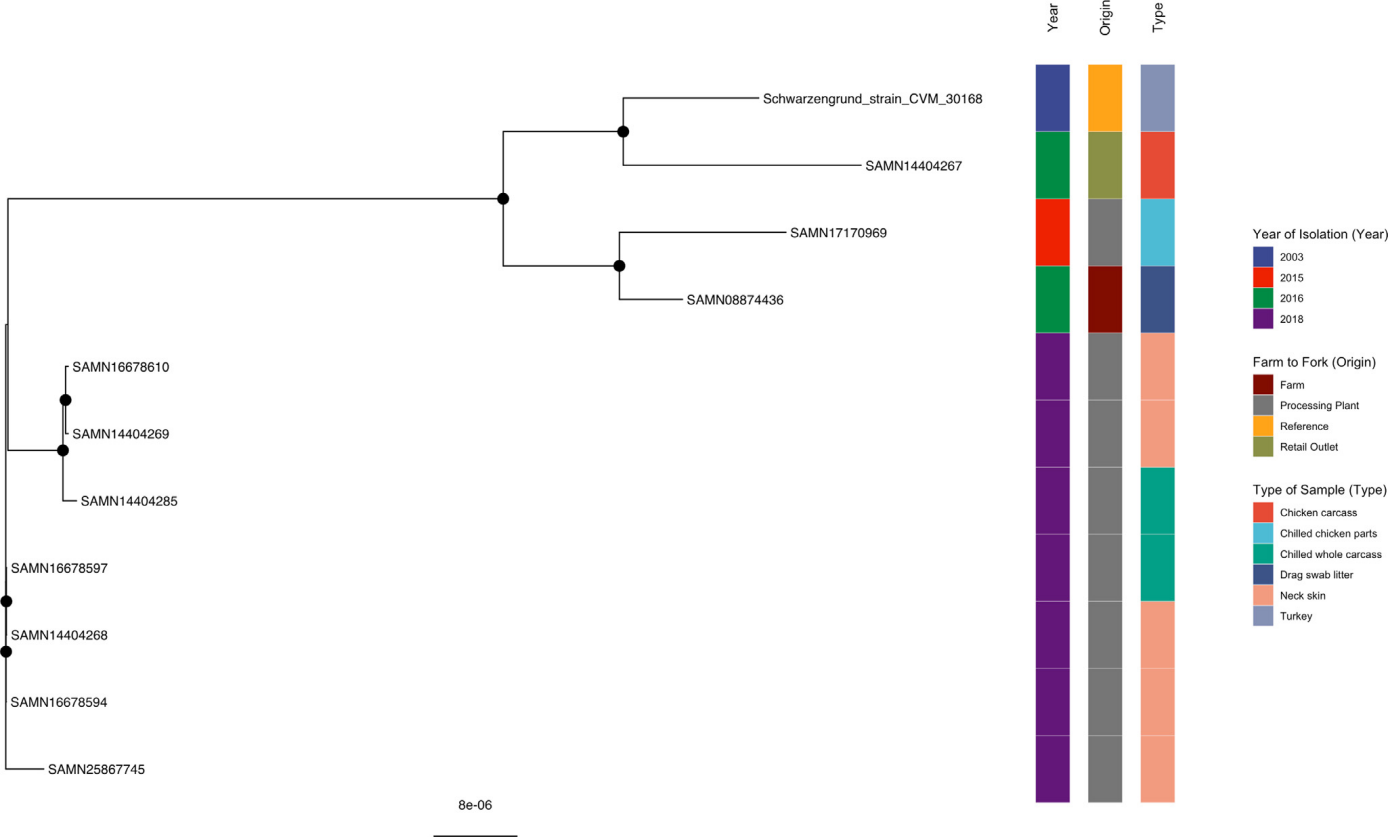
Phylogenetic analysis of *S.* Schwarzengrund. Maximum-likelihood phylogeny of *S.* Schwarzengrund is shown, calculated using a core genome alignment with IQ-TREE v.2.1.2 and 1,000 bootstraps specified. A black dot on the node indicates bootstrap values >70%.

Strain SAMN14404267, isolated from a retail outlet in this study (2016), was clustered with a USA reference strain (2003) as well as 2 other Brazilian strains from a farm (2016) and processing plant (2015) (Figure 8, Supplementary Table 8) and supported by a bootstrap value of 100%. All the Schwarzengrund strains isolated from neck skins and chilled whole carcasses that originated from Plants B and D were clustered together and well-supported except for SAMN25867745 (neck skin, Plant D), which was unclustered with the other strains in this tree.

#### *S*. Senftenberg

**Figure 9:**
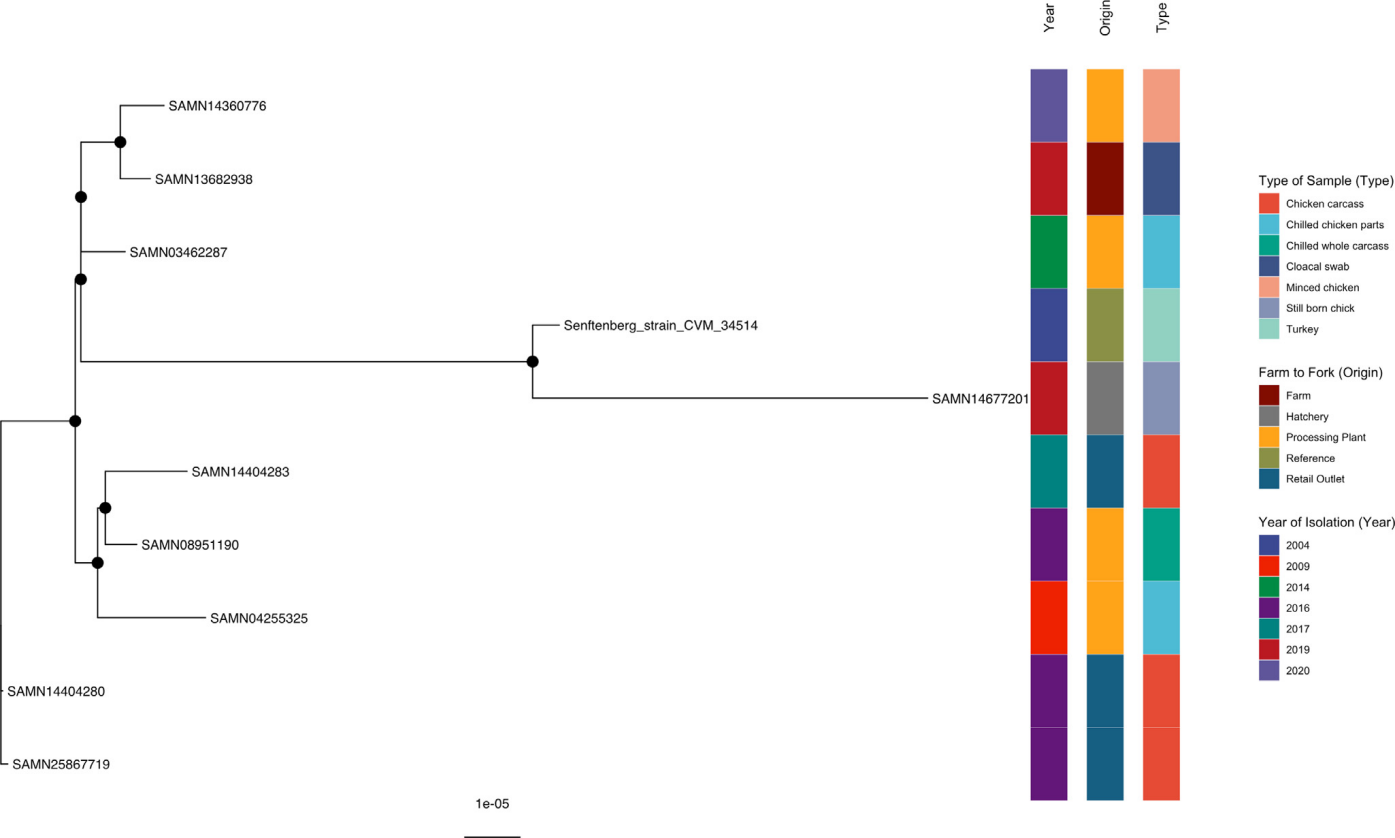
Phylogenetic analysis of *S.* Senftenberg. Maximum-likelihood phylogeny of *S.* Senftenberg is shown, calculated using a core genome alignment with IQ-TREE v.2.1.2 and 1,000 bootstraps specified. A black dot on the node indicates bootstrap values >70%.

The only Senftenberg serovar (SAMN14677201) isolated at hatcheries in our study was clustered with four international strains (Senftenberg_strain_CVM_34514, SAMN03462287, SAMN13682938, and SAMN14360776) (Figure 9, Supplementary Table 9). This tree is included and supported by a bootstrap value of 100%. Of significance is the clustering of SAMN14677201 (stillborn chick, 2019) in this study with SAMN13682938, a cloacal swab sample detected at a farm in the United States in 2019, and SAMN14360776, isolated from minced chicken at a processing plant in the United States (2020), well-supported branches. In addition, one of the retail outlet strains, SAMN14404283 in the current study, showed well-supported clustering with SAMN08951190, a processing plant strain isolated in Brazil in 2016. The other retail outlet strains in this study were unclustered to one another and other members of this tree.

## DISCUSSION

This is the first documented phylogenetic study conducted using WGS in the poultry industry (broiler production, processing, and retailing) in Trinidad and Tobago and the Caribbean region at large. Whole-genome sequencing analysis has been used to investigate genetic characteristics and phylogenies among *Salmonella* isolated from different origins, such as humans, food, animals, and environmental samples elsewhere (Lee et al., 2015; Toro et al., 2016; Pearce et al., 2018; Pightling et al., 2018).

Our WGS analyses revealed well-supported clustering of isolates recovered within farms (*S.* Infantis), within processing plants (Anatum, Infantis, and Schwarzengrund), and between processing plants and retail outlets (Javiana and Kentucky), along the farm-processing plant-retail outlet chain (Albany). Additionally, Intra-Caribbean (Kentucky; Barbados) and international associations (United States, Brazil, China, and Peru) clustering of *Salmonella* serovars were demonstrated in the current study. These findings are significant as they highlight the well-supported clustering of *Salmonella* serovars within and between different stages of the broiler production chain in the country, regional, and international sources, respectively, having public health significance, the serovars studied have been implicated in human salmonellosis.

All fertile broiler-hatching eggs were imported from 2 companies in the USA. While all imported fertile hatching eggs were negative for *Salmonella* (Khan et al., 2022a), *S.* Kentucky strains were detected at the 2 hatcheries sampled (operated by Processor A and B); therefore, it was essential to determine the relatedness of these strains. Significantly, the Kentucky strains detected at the respective hatcheries were not clustered together, a finding that could be attributed to different sources of the imported fertile hatching eggs. At variance with the findings in the current study, Kim et al. (2007) reported that *S.* Enteritidis strains isolated from different hatcheries in Korea exhibited the same PFGE types. That indicated cross-transmission among hatcheries was possibly due to sharing the same source of eggs, a practice common at local hatcheries in this study. However, a serotype Kentucky strain detected at the hatchery operated by Processor A showed well-supported clustering with retail outlet strains detected in the current study and a retail outlet strain recovered in Barbados. It is known that day-old chicks in Barbados are exported to other Caribbean countries, thereby potentially disseminating this strain. Another important finding was the well-supported clustering exhibited among most Kentucky strains detected at the hatchery operated by Processor B to a drag swab sample in the United States. Based on this, it can be hypothesized that this strain was imported into the country through fertile broiler hatching eggs. This is supported by the findings of a recent study that implicated international trade as the source of global *Salmonella* dissemination (Li et al., 2021). *S.* Kentucky is a well-known broiler-associated serovar with limited public health significance; therefore, its detection and relatedness are essential to the poultry industry, especially with the increasing development of AMR.

At the farm level in this study, well-supported clustering among *S.* Albany and *S.* Infantis strains (mostly cloacal swabs) detected at different farms operated by the same and different processors was observed. While all broiler farms in the country received chicks from processors to which they are attached, it is unclear why the clustering of strains across farms was observed since they were in different counties. A possibility exists of spreading the serovar across poultry farms by pests, such as pigeons, which were a common pest found on most farms, and have been implicated in *Salmonella* dissemination (Trampel et al., 2014).

Within processing plants, reliable clustering was observed among *S.* Anatum (Plant B), and Infantis (Plant A) strains detected on the same sampling day at the respective plant. Whereas *S*. Schwarzengrund strains detected on different sampling days at different plants (Plant B and D) exhibited well-supported clustering among each other. This finding was expected as it is common at processing plants to receive birds that would have originated from farms operated by different processors due to chicken shortages at some smaller processors. Mendonca et al. (2020) reported the persistence of 7 strains of serovar Infantis in environmental swabs taken at broiler farms over 5 months and attributed this to biofilm formation. They also isolated homologous *Salmonella* strains from environmental swabs of the farm and the meat matrices at the processing plant, which were suggestive of negligence in biosafety protocols in the production line, which resulted in contaminated carcasses and meat cuts. Additionally, the authors detected another cluster of strains that included various samples from within the processing plant, which indicated the persistence of the strain within the plants and subsequent contamination of chickens during processing. The findings of persistence in their study (Mendonça et al., 2020), agree with our current research where serovars Anatum, Infantis, and Schwarzengrund were detected in chilled whole, chilled chicken parts, and neck skin samples at various periods during the sampling day. These findings suggest ineffective control strategies for *Salmonella* elimination or biofilm formation at the processing plants. A study conducted by Hyeon et al. (2021) on *S.* Enteritidis strains in South Korea reported the persistence of genetically related *Salmonella* strains in carcasses at processing plants and in the environment of slaughterhouses (evisceration room, and chilling rooms), which were suggestive of the presence of resident strains in those environments. These findings agree with our detection of genetic relatedness of *Salmonella* isolates within processing plants. In addition, the detection of well-supported clustering among isolates of serovars Infantis recovered from broiler farms (cloacal swabs, drinking water, and drag swabs of litter) to isolates of the same serovar recovered from the processing plant where the broilers were slaughtered concurs with a published report (Mendonça et al., 2020).

Strains of Javiana and Kentucky detected at retail outlets exhibited reliable clustering. Serovar Javiana strains detected at retail outlets (pluck shops) showed reliable clustering to processing plant strains (Plant B), which could be attributed to the fact that chickens sold at pluck shops could have originated from the same farm (with resident strains). It is common practice that chickens from farms are either processed at processing plants or sold to the ‘live market’ where they are slaughtered at pluck shops. Therefore, chickens sold at pluck shops and those processed at Plant B might have originated from the same farm with resident strains. Strains of Kentucky detected at the same pluck shop, and different supermarkets exhibited reliable clustering. Like the findings at pluck shops, chickens sold at supermarkets were processed at processing plants. Therefore, chickens sold at various supermarkets might have originated from the same farm or processing plant with resident Kentucky strains. It should be highlighted that retail outlet and processing plant sample collection was conducted in 2016–2017 and 2018, respectively; therefore, the presence of resident strains is a plausible cause.

Dissemination to downstream stages of the broiler production chain, persistence, and difficulty of elimination from the environment has been attributed to biofilm-formation ability (Nesse et al., 2003; Prunić et al., 2016). Several phylogenetic studies conducted on *S.* Kentucky, Typhimurium, and Schwarzengrund strains concluded that animals are reservoirs of *Salmonella* spp. and a source of human and environmental contamination (Eguale et al., 2017; Pornsukarom et al., 2018). The detection of strains of international relevance in this study agrees with the statement that global dissemination of *Salmonella* spp. is facilitated by travelers, imports, and exports of livestock and livestock products (Ashton et al., 2017). Internationally related *S.* Infantis strains detected in the Galapagos Islands (Burnett et al., 2021) showed close relatedness to strains in the United States, Peru, and Ecuador (Tate et al., 2017; Brown et al., 2018). Similarly, in the current study, well-supported clustering was observed between our strains of serovar Infantis and those recovered in the United States, Brazil, China, and Peru. Even though the years of detection varied, it may be attributable to their persistence at hatcheries (through imported fertile hatching eggs), farms and processing plants due to biofilm formation, and inadequate sanitation protocols at the different localities. Of major significance in this study was the clustering of serovar Kentucky strains recovered from various sources within a hatchery to a farm isolate detected in the United States, considering that all fertile hatching broiler eggs were imported into Trinidad and Tobago, and originated from the United States. Even though 450 imported fertile hatching eggs tested were all *Salmonella* negative, the detection of Kentucky strains from various sources within hatcheries has the potential to proliferate and disseminate rapidly at hatcheries, to farms, processing plants, and retail outlets, given the conditions maintained in incubators and hatchers (Mendonça et al., 2020). The regional clustering of *S.* Kentucky strains between Trinidad and Tobago and Barbados could be explained by the practice of exportation/importation of poultry livestock in the Caribbean.

Of food safety and clinical significance was that 2 serovars (Infantis and Enteritis) isolated locally from human gastroenteritis cases during the period of our cross-sectional studies exhibited well-supported clustering with our isolates from the broiler industry (neck skins, chilled whole carcasses, chilled chicken parts). The possibility exists of human exposure through the consumption of improperly cooked chicken or chicken products, which originated from the processing plants in the country.

Phylogenetic analyses proved helpful in comparing the genetic sequences among strains of *Salmonella* to investigate their homology and their relatedness using the demographic and epidemiological data generated from the cross-sectional studies conducted at 3 levels (production, processing, and retailing) of the broiler industry in the country. Well-supported clustering among isolates recovered within hatcheries, farms, and processing plants, between processing plants and retail outlets, farm-processing plant-retail outlets, inter-Caribbean (Kentucky; Barbados), and international associations (United States, Brazil, China, and Peru) were highlighted in this study. In addition, to food safety and clinical significance was the fact that strains belonging to the serovar Infantis and Enteritidis, isolated locally from human gastroenteritis cases during the period of our cross-sectional studies, exhibited well-supported (>70% bootstrap) clustering with our isolates from the broiler industry (neck skins, chilled whole carcasses, chilled chicken parts). This suggests that the human clinical *S.* Infantis and *S.* Enteritidis strains isolated in 2019 might have originated from locally produced chickens based on the high bootstrap values of the respective strains. Therefore, the possibility exists of human exposure through the consumption of improperly cooked chicken or chicken products that originated from the processing plants in the country.

## Appendix Supplementary materials

Image, application 1
